# Metabolomic Profiling Screening for Molecular Level Associations Related to Cashmere Quality Related Phenotypic Types in Liaoning Cashmere Goats

**DOI:** 10.3390/genes17101298

**Published:** 2026-10-18

**Authors:** Nan Zhou, Meng Sun, Huiying Liu, Chunqiang Wang, Huaizhi Jiang

**Affiliations:** 1 College of Animal Science and Technology, Jilin Agricultural University, Changchun 130118, China; 18629923164@163.com (N.Z.); 13845921316@163.com (H.L.); 2 Department of Animal Science, College of Agriculture, Jinzhou Medical University, Jinzhou 121000, China; 13252884157@163.com (M.S.); wcqworld@126.com (C.W.); 3 Liaoning Provincial Key Laboratory of Animal Product Quality and Safety, Jinzhou Medical University, Jinzhou 121000, China

**Keywords:** Liaoning cashmere goat, metabolomics, cashmere quality, nominally associated candidate metabolites, skin tissue

## Abstract

**Background/Objectives:** Distinct cashmere-related traits are observed between long cashmere short wool (LC-SW) and short cashmere long-hair (SC-LW) Liaoning cashmere goats. Yet metabolic differences associated with these phenotypes remain poorly characterised. This study explored skin metabolic divergence between two specific farm-raised maternal families that consistently exhibit LC-SW and SC-LW phenotypes, providing preliminary references for improving cashmere traits. Considering experimental limitations, phenotype is confounded by maternal family genetic background; therefore, this work does not aim to uncover universal mechanisms underlying breed-wide LC-SW/SC-LW subtypes. **Methods:** 120 healthy adult female Liaoning cashmere goats from two independent maternal families were used for phenotypic measurement. Among them, six animals from each family (total *n* = 12) were randomly selected for untargeted metabolomic analysis of skin tissue using GC-TOF-MS. **Results:** The two maternal families differed significantly in body weight, cashmere yield and cashmere fibre length (*p* < 0.05), whereas cashmere fibre diameter showed no significant difference. In total, 89 nominally associated candidate metabolites were mapped to the following KEGG pathways: amino acid biosynthesis, ABC transporters, and central carbon metabolism in cancer (KEGG database annotations for core carbon-related metabolites; no cancer-related physiology is implied). The LC-SW family showed higher relative abundances of metabolites associated with glycolysis/TCA-related metabolism, as well as multiple amino acid-related metabolites. These energy-related and keratin precursor-related metabolites exhibit higher relative abundance in the LC-SW-related maternal family. The SC-LW family exhibited reduced levels of γ-aminobutyric acid and glutamate, indicating metabolite-level differences that warrant further investigation into links with cashmere length traits. **Conclusions:** This study characterises skin metabolomic profiles of two phenotype-divergent maternal families. These findings offer preliminary data for cashmere trait research, but cannot be generalised to all LC-SW and SC-LW goats. Further validation using materials from multiple unrelated families is required.

## 1. Introduction

The Liaoning cashmere goat is a well-known dual-purpose breed for cashmere and meat production in China, characterised by high cashmere yield, moderate fibre diameter, robust body conformation, good adaptability, and reliable genetic stability [1]. It is widely recognised as a valuable indigenous genetic resource and has been frequently used to improve the genetic performance of low-producing goat populations across China [2]. Cashmere fibre serves as a premium textile raw material; key quality traits including fibre length, fineness, strength and lustre largely determine its commercial value [3]. Accordingly, uncovering the physiological and molecular mechanisms governing cashmere quality formation is critical for promoting sustainable development of the cashmere industry.

Liaoning cashmere goats are mainly distributed in Gai Zhou and surrounding regions of Liaoning Province. Long-term artificial selective breeding has generated several distinct maternal families, such as high-yield, multi-fibre, and high-quality phenotypic groups [4]. Notably, two phenotypically distinct maternal family variants, namely long cashmere short wool (LC-SW) and short cashmere long wool (SC-LW), have been observed in the Wafangdian population. At present, knowledge of these two variants largely derives from field observations and simple phenotypic measurements, while the intrinsic molecular mechanisms underlying their divergent cashmere quality phenotypes remain poorly characterised. This knowledge gap restricts the development of molecular marker-assisted breeding for cashmere-related traits [5].

Cashmere fibres originate from secondary hair follicles in the skin. Cashmere quality is collectively shaped by hair follicle development, cyclic regeneration, and the local skin microenvironment [6]. Skin metabolic microenvironments, involving amino acid supply, energy metabolism, and antioxidant status, provide the raw materials and energy for follicle cell proliferation and differentiation. Moreover, metabolic fluctuations can indirectly modify the physicochemical properties of fibres by regulating oxidative stress and cell membrane fluidity [7]. Therefore, phenotypic divergence in cashmere quality may fundamentally stem from differences in small-molecule metabolite profiles within skin tissues.

With advances in mass spectrometry-based high-throughput omics platforms, metabolomics has become a powerful tool for dissecting complex economic traits in livestock [8]. Metabolomics directly profiles small-molecule metabolites and reflects the real-time physiological and biochemical status of organisms. It has been widely used to explore the molecular basis of meat, milk, and hair fibre quality in domestic animals. Previous metabolomic studies have identified metabolites correlated with hair follicle cyclic activity in cashmere goats, highlighting the importance of skin metabolic processes for fibre growth [9]. Notably, the work of Ma et al. characterised metabolic alterations accompanying hair follicle cycle transitions in a single goat population. Unlike their research design, our study focuses on intrinsic metabolic divergence between two maternal families exhibiting distinct cashmere-related phenotypes (LC-SW and SC-LW) at the same cashmere anagen phase, aiming to elucidate the metabolic basis of phenotypic differences in cashmere quality rather than follicle-cycle-related metabolic variation.

Nevertheless, metabolomic evidence explaining phenotypic differences between LC-SW and SC-LW Liaoning cashmere goat phenotypes remains lacking. In the present study, we systematically measured core cashmere quality phenotypes of LC-SW and SC-LW goats raised under identical farm conditions. Skin samples collected during the cashmere anagen phase were subjected to untargeted metabolomics using GC-TOF-MS. Nominally associated candidate metabolites and corresponding KEGG mapping pathways were identified via bioinformatic analysis.

Metabolomics captures intermediate functional molecular phenotypes that bridge genetic background and observable complex agronomic traits. Even without genome-wide marker data, characterising metabolite-level divergence can generate testable candidate clues for later genetic mapping and quantitative trait locus studies in cashmere goats. This study aimed to characterise metabolite differences associated with the contrasting phenotypes. This study provides a theoretical basis and candidate metabolic markers to support molecular breeding and nutritional regulation of cashmere traits in Liaoning cashmere goats.

## 2. Materials and Methods

### 2.1. Experimental Animals and Sample Collection

These were obtained from the Wafangdian Animal Husbandry Extension Centre (the same farm in Liaoning, China). The LC-SW and SC-LW-related groups were derived from two independent, non-inbred maternal families maintained on this farm. Non-inbred status was confirmed by reviewing the farm’s pedigree breeding records. Three-generation pedigree information for each selected animal was retrospectively reviewed, and no close-relative mating events were documented within three generations. It should be noted that this evaluation relies on farm-maintained pedigree files rather than genome-derived inbreeding coefficients. In this farm-breeding context, a maternal family is defined as the progeny descending from a common foundation dam. Multiple unrelated sires were used for mating across generations within each maternal family; animals within one maternal family share the common maternal ancestor but do not share a unified paternal lineage. These two maternal families have consistently exhibited LC-SW and SC-LW-related phenotypic characteristics across generations under identical feeding and management conditions, according to long-term farm observations. (Notably, cashmere-related phenotype and maternal family genetic background cannot be fully disentangled in the present experimental design).

Inclusion criteria: (1) Adult female Liaoning cashmere goats classified as either LC-SW or SC-LW phenotype according to long-term on-farm phenotypic observation; (2) clinically healthy without skin lesions and infectious diseases; (3) similar age, 1.5 years old; (4) reared under identical feeding and husbandry management conditions.

Exclusion criteria: (1) Individuals suffering from skin injury, parasitic infestation or other systemic diseases; (2) goats with ambiguous phenotypic classification; (3) animals receiving any drug intervention within three months before sample collection.

For phenotypic evaluation, cashmere samples were collected from 60 randomly selected goats within each population (LC-SW: *n* = 60; SC-LW: *n* = 60; total *n* = 120) during the cashmere growth phase (September). The specific procedures were as follows: a 5 cm × 5 cm area was marked on the right scapular region of each goat. A ruler was placed perpendicular to the skin surface and pressed against the base of the cashmere tufts to measure cashmere length directly. Multiple replicates of cashmere fibre length were measured for each goat. For each animal, the arithmetic mean of these replicates was calculated and used as its representative fibre-length phenotypic value for subsequent analysis. After measurement, curved scissors were used to clip the cashmere from the marked area as close to the skin as possible. The collected cashmere was then stored in a sealed bag under natural conditions, and each bag was labelled for subsequent measurement of cashmere fibre diameter.

Following phenotypic measurement of these 120 goats, a subset of six animals was randomly selected from each population for skin tissue sampling for GC-TOF-MS metabolomics analysis (LC-SW: *n* = 6; SC-LW: *n* = 6; total skin samples: *n* = 12). TOF-MS metabolomics analysis (LC-SW: *n* = 6; SC-LW: *n* = 6; total skin samples: *n* = 12). All 120 goats used for phenotypic evaluation were 1.5 years old. The 12 animals selected for metabolomic analysis were a subset of this cohort and also aged 1.5 years. Parity and body condition score were not recorded during sample collection, which is acknowledged as a limitation of the present study. All individuals are non-pregnant and non-lactating goats; All experimental goat feed and feeding management are consistent.

After cashmere fibre collection, prior to skin biopsy collection, local anaesthesia was administered at the sampling site using lidocaine hydrochloride injection to alleviate pain and discomfort in goats. The skin biopsy was obtained from the upper edge of the scapula using a biopsy punch with a diameter of 1 cm after local anaesthesia took effect. The 1 cm diameter biopsy punch penetrated full-thickness skin (epidermis, dermis and superficial subcutaneous layer) to ensure complete capture of secondary cashmere hair follicle units. The samples were immediately frozen in liquid nitrogen and stored at −80 °C for subsequent use. After skin biopsy, the wound was disinfected locally. The goats were monitored daily for signs of inflammation, swelling, redness or wound dehiscence for one week post-sampling. No adverse complications were observed in any animal during the monitoring period.

After natural drying, the cashmere samples were tested, cleaned, and processed. The diameter of cashmere fibres was measured using an N-107CCD wool fineness tester (Ningbo Yongxin Optics Co., Ltd., Ningbo, China). (For each goat, thirty cashmere fibres were randomly selected and observed under the microscope across 3 independent microscope fields. Fibre diameter values were measured, and the arithmetic mean was taken as the individual representative fibre diameter phenotype).

Data on body weight and cashmere yield of the goats were provided by the farm. All methods and procedures were performed in accordance with the relevant guidelines established by the Ministry of Agriculture of the People’s Republic of China. (Body weight of each goat was measured using a calibrated electronic livestock scale before combing. Cashmere yield was gravimetrically determined by manually collecting and weighing total cashmere obtained during the annual cashmere combing procedure for each individual animal). Ethical approval for this study was obtained from the Ethics Committee of Jilin Agricultural University.

### 2.2. Metabolomics Analysis of Skin Tissue

For metabolite extraction, 25 mg ± 1 mg of each skin sample was transferred into a 2 mL EP tube, to which 500 μL of pre-cooled methanol-chloroform extract (3:1, *v*/*v*) containing 10 μL ribitol was added. Samples were vortexed for 30 s, homogenised using a ball mill at 40 Hz for 4 min, and then subjected to 5 min of sonication in an ice? water bath; this homogenization-sonication cycle was repeated four times. After centrifugation at 4 °C and 11,000 rpm for 15 min, 200 μL of supernatant was collected from each sample. Quality control (QC) samples were prepared by pooling 50 μL supernatant aliquots taken from every individual sample. Following vacuum concentration, 30 μL of 20 mg/mL methoxyamination hydrochloride (dissolved in pyridine) was added for derivatisation, and samples were incubated at 80 °C for 30 min. Subsequently, 40 μL of BSTFA reagent (1% TMCS, *v*/*v*) was applied, and samples were incubated at 70 °C for 1.5 h before gradual cooling to room temperature. FAMEs (dissolved in chloroform, 5 μL) were added to each QC sample, and all prepared samples were analysed by GC-TOF-MS.

GC-TOF-MS analysis was performed using an Agilent 7890 gas chromatograph coupled with a time-of-flight mass spectrometer. The system utilised a DB-5MS capillary column. 1 μL aliquot of the sample was injected in splitless mode. Helium was used as the carrier gas; the front inlet purge flow was 3 mL min^−1^, and the gas flow rate through the column was 1 mL min^−1^. The initial temperature was kept at 50 °C for 1 min, then raised to 310 °C at a rate of 10 °C min^−1^, then kept for 8 min at 310 °C. The injection, transfer line, and ion source temperatures were 280, 280, and 250 °C, respectively. The energy was −70 eV in electron impact mode. The mass spectrometry data were acquired in full scan mode with the m/z range of 50~500 at a rate of 12.5 spectra per second after a solvent delay of 6.35 min.

For data pre-processing, an aliquot of 20 μL ribitol internal standard solution (0.2 mg/mL) was added to each sample. Raw peak-area intensities of each feature were normalised using the peak area of the ribitol internal standard. Sample injection order was fully randomised; pooled quality-control (QC) samples prepared by mixing equal aliquots from all experimental samples were repeatedly inserted among real-sample injections to monitor analytical drift and batch-to-batch stability. Peak-table features with RSD > 30% across QC samples were discarded. For remaining features, missing values originating from low-abundance signals were handled by filtering out features with >20% missing rate across all samples; remaining valid missing entries were imputed with half of the minimum observed value of that feature. Prior to multivariate statistical analysis (PCA and OPLS-DA), Normalised peak-intensity data were log10-transformed and unit-variance (UV)- scaled. Was metabolite identification performed by matching experimental mass spectra and retention-index (RI) values against the in-house built metabolite spectral library. The mass spectrum match threshold was set to ≥700 (0–1000 scale), and the retention index tolerance was set to ±50 RI units. Metabolites that met both spectral match score and RI-tolerance criteria were assigned as level-2 confidence identifications according to MSI (Metabolomics Standards Initiative) reporting standards. Features that failed these criteria were kept as unknown, unnamed peaks and excluded from downstream biological interpretation. Significantly altered metabolites were subsequently subjected to KEGG pathway mapping analysis (Kyoto Encyclopedia of Genes and Genomes, Kyoto Encyclopedia of Genes and Genomes, http://www.genome.jp/kegg/ (accessed on 6 July 2025) [10]. No formal multiple testing FDR correction was performed.

### 2.3. Statistical Analysis

Phenotypic indicators (body weight, cashmere yield, cashmere fibre-related traits) between two maternal families were compared using an independent-samples Student’s *t*-test in SPSS 26.0 software, after checking normality and homogeneity of variance. *p* < 0.05 was considered the threshold for phenotypic significance.

Metabolomics-related multivariate analyses, including principal component analysis (PCA) and orthogonal partial least squares discriminant analysis (OPLS-DA), were conducted for global metabolomic profiling. Nominally associated candidate metabolites were screened by Student’s *t*-test (*p* < 0.05) combined with OPLS-DA VIP > 1. Formal false-discovery-rate multiple-testing correction was not performed in this exploratory study.

Pairwise metabolite-to-metabolite correlation analysis was performed using Pearson correlation based on normalised metabolite intensities across the 12 skin metabolomic samples. In total, pairwise correlations were calculated for all nominally associated candidate metabolites. Given the very limited sample size (*n* = 12), correlation coefficients are statistically unstable. Therefore, the correlation heatmap is presented for descriptive visualisation purposes only. No multiplicity testing correction was applied, and no inferential statistical claims about correlation significance are drawn from this heatmap. Hierarchical clustering was performed based on the correlation matrix using Ward’s clustering algorithm. All correlation and clustering analyses were performed in R.

We performed Benjamini-Hochberg FDR correction for multiple testing across all detected metabolites. Very few metabolites passed the FDR significance threshold (q < 0.05). Therefore, metabolites with nominal *p* < 0.05 combined with VIP > 1 from OPLS-DA are reported as nominally associated candidate metabolites rather than statistically confirmed differential metabolites. These associations are exploratory and require independent validation in larger cohorts.

KEGG pathway mapping was performed by assigning candidate metabolites to KEGG pathway entries. Due to the lack of a well-annotated skin metabolome reference set for Liaoning cashmere goats, formal statistical overrepresentation analysis with KEGG pathway mapping statistics and *p*-values was not conducted.

## 3. Results

### 3.1. Comparison of Cashmere Fiber Phenotypic Traits in Cashmere Goats

Phenotypic comparison was performed on 120 experimental goats (60 animals per maternal family). Phenotypic indicators, including body weight, cashmere yield, cashmere fibre length, and cashmere fibre diameter, were compared between the two maternal families with LC-SW and SC-LW-related phenotypes. The LC-SW-related maternal family showed significantly higher body weight, cashmere yield, and cashmere fibre length compared with the SC-LW-related maternal family(*p* < 0.05). No significant inter-family difference was detected in cashmere fibre diameter (*p* > 0.05). (Table 1). Twelve goats (6 per family) were randomly sampled from this 120-animal cohort for subsequent skin metabolomic profiling.

### 3.2. Results of Skin Metabolomics Analysis

#### 3.2.1. Quality Assessment of Metabolite Data

**Figure 1:**
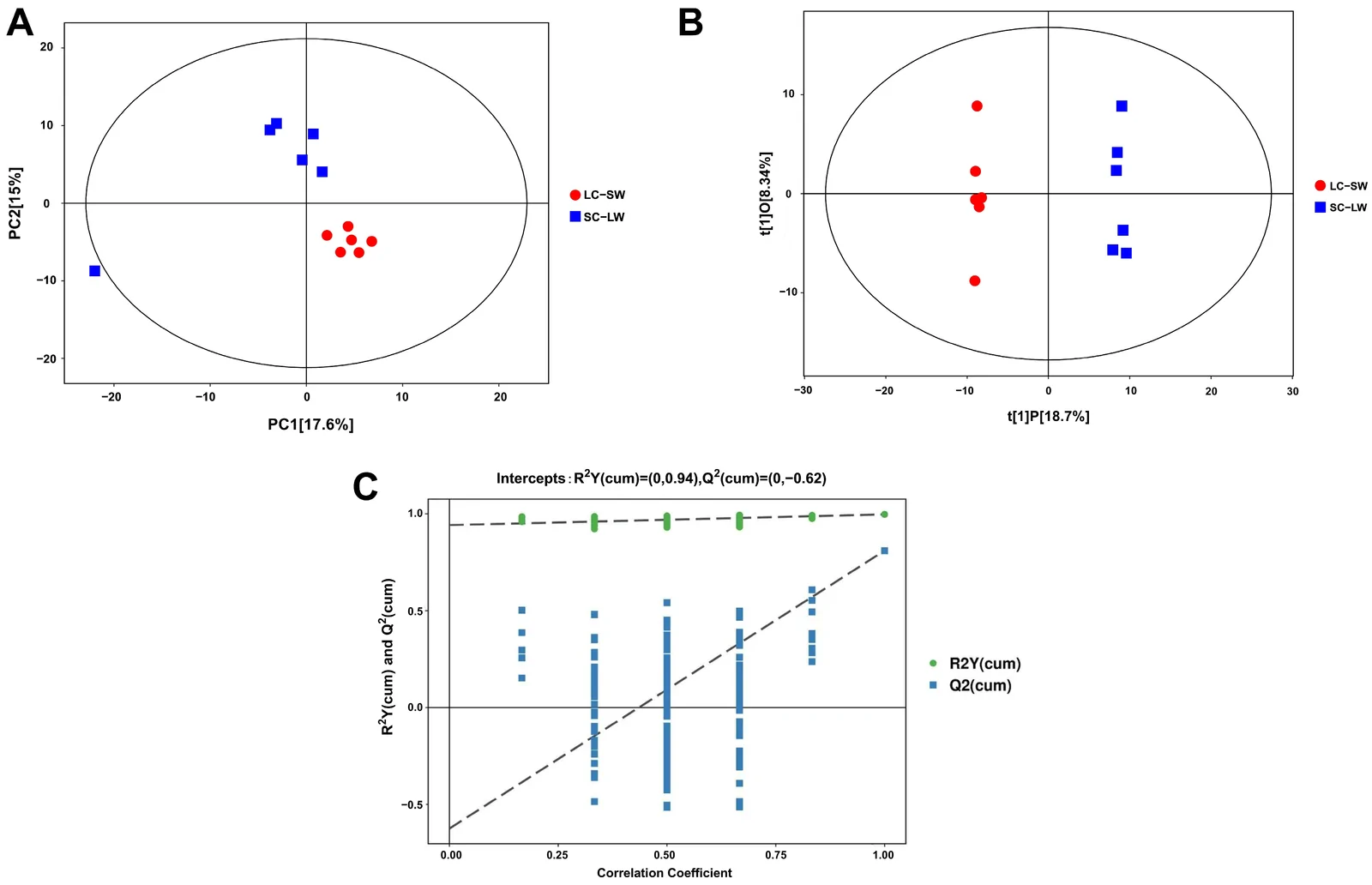
Quality assessment and control of metabolomics data. (**A**) PCA score plot of skin metabolism profiles. The horizontal axis (PC1) and the vertical axis (PC2) represent the scores of the first and second principal components, respectively. Each scatter point represents a sample, with its colour and shape indicating different groups. The samples roughly fall within the 95% confidence interval (Hotelling’s T-squared ellipse). (**B**) OPLS-DA score plot for group discrimination. The horizontal axis, t[1]P, represents the predicted principal component scores of the first component, displaying the between-group variation among samples. The vertical axis, t[1]O, represents the orthogonal principal component scores, showing the within-group variation. Each scatter point represents a sample, with its shape and colour indicating the different experimental groups. (**C**) Permutation test result (*n* = 200 permutations) for OPLS-DA model to evaluate over-fitting. For OPLS-DA modelling, the cumulative R^2^X value was reported to reflect the explained variation. For the OPLS-DA model, R^2^X (cumulative), R^2^Y (cumulative) and Q^2^ values were calculated. A permutation test with 200 permutations was performed to judge whether the OPLS-DA model suffered from over fitting. Model diagnostics together with score-plot visualisation were used to evaluate group separation.

Principal component analysis (PCA) was applied to determine the degree of sample separation and clustering among the cashmere goat metabolites. Meanwhile, orthogonal partial least squares discriminant analysis (OPLS-DA) was performed to obtain more reliable metabolite differences between the groups. The OPLS-DA score plot revealed a clear separation between the two maternal families. Given the small sample size (*n* = 6 per maternal family), this trend should be interpreted with caution. (Figure 1A). PC1 and PC2 explained 17.6% and 15.0% of the total variance, respectively, indicating a clear systematic difference between the two groups, mainly along the PC1 direction. This suggests that the two groups have distinguishable overall characteristics in their metabolite or expression profiles. OPLS-DA showed that the predictive component t [1] And the orthogonal component P together built the model, in which the predictive component explained 18.7% of the between-group variance (Figure 1B), indicating that the model captured significant signals of differences between the groups.

The OPLS-DA model yielded cumulative Q^2^ = (0, −0.62), cumulative (R^2^Y) = (0, 0.94). A permutation test with 200 random permutations was performed to assess over-fitting. The intercept of Q^2^ from permutation testing was −0.62, indicating no severe over-fitting of the OPLS-DA model (Figure 1C).

#### 3.2.2. Analysis of Nominally Associated Candidate Metabolites

**Figure 2:**
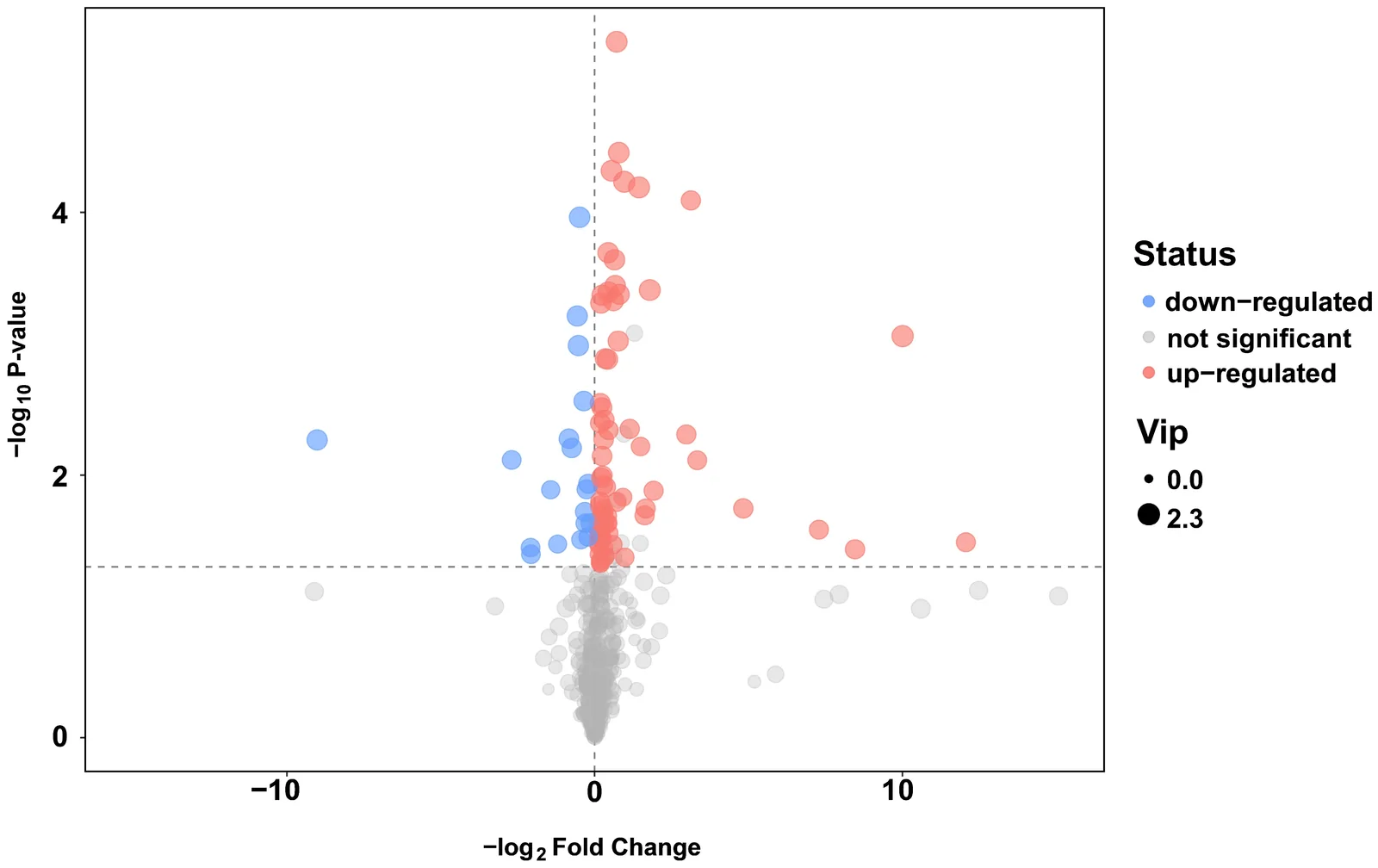
Volcano plot for screening nominally associated candidate metabolites. In the volcano plot, each point represents a metabolite. The horizontal axis denotes the log_2_-transformed fold change of each substance in the group comparison. In contrast, the vertical axis represents the negative logarithm (base 10) of the *p*-value from Student’s *t*-test. The size of each scatter point corresponds to the VIP value from the OPLS-DA model, with larger points indicating higher VIP values. The colour of the points indicates the final screening outcome: significantly up-regulated metabolites are shown in red, significantly down-regulated metabolites in blue, and non-significant metabolites in grey. Note: Volcano plot showing metabolite differences between two phenotypic groups. The horizontal dashed line corresponds to nominal unadjusted *p* < 0.05. Displayed *p*-values are nominal without FDR correction, and adjusted q-values are available in Supplementary Table S1. (*n* = 6 per group). Results are exploratory and should be interpreted with caution.

**Figure 3:**
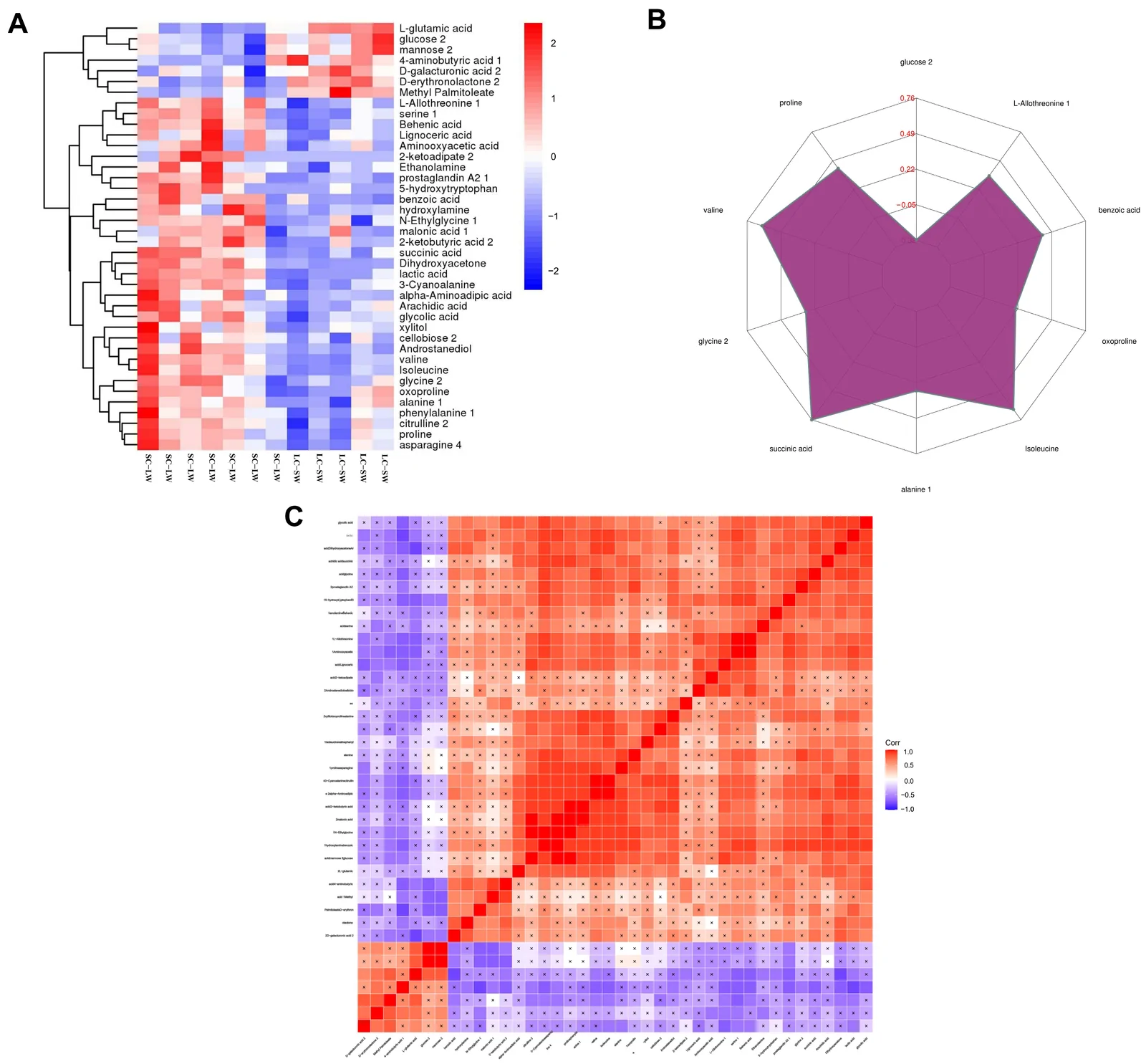
Analysis of nominally associated candidate metabolites. (**A**) Hierarchical cluster analysis of nominally associated candidate metabolites: The horizontal axis represents the different experimental groups, while the vertical axis lists the nominally associated candidate metabolites identified in the comparison. The color of each tile (cell) indicates the relative expression level of the corresponding metabolite: red denotes high abundance, and blue denotes low abundance. (**B**) Radar chart analysis of nominally associated candidate metabolites. For these metabolites, fold-change ratios were calculated from quantitative values and log_2_-transformed; these log_2_ fold-change values are annotated in red font within the chart. Each grid line represents a specific fold-change magnitude. The purple shaded polygon connects log_2_ fold-change values for each metabolite to visually illustrate abundance trends. Note that this radar plot only presents log_2_ fold-change; VIP values from the OPLS-DA discriminant model are provided in the main text and Supplementary Table S1. (**C**) Correlation heatmap analysis for nominally associated candidate metabolites. Both axes represent nominally associated candidate metabolites. The tile colour corresponds to Pearson correlation coefficient r (red = positive correlation, blue = negative correlation; darker colour indicates larger absolute r value). This heatmap serves only descriptive visualization. Due to small sample size *n* = 12, correlation estimates are inherently unstable; no inferential significance testing conclusions are drawn from this plot. ^x^ *p* < 0.05.

Multivariate statistical analysis was used to analyse the data. Metabolites with a Student’s *t*-test *p*-value < 0.05 and a variable importance in projection (VIP) > 1 from the first principal component of the OPLS-DA model were considered nominally associated candidate metabolites. A total of 89 nominally associated candidate metabolites were identified (volcano plot, Figure 2, Supplementary Table S1), of which 70 were upregulated and 19 were downregulated. The nominally associated candidate metabolites were ranked in descending order according to their VIP values. The top 10 known nominally associated candidate metabolites were: Dihydroxyacetone, lactic acid, valine, Isoleucine, serine, 4-aminobutyric acid, L-glutamic acid, Behenic acid, succinic acid, and benzoic acid. Among these metabolites, 4-aminobutyric acid and L-glutamic acid were significantly downregulated in the SC-LW group compared to the LC-SW group. Hierarchical clustering analysis of the nominally associated candidate metabolites visually showed changes in the relative abundance or expression levels of various metabolites between the two groups (Figure 3A). For example, L-glutamic acid and glucose showed clear downregulation in the SC-LW group, suggesting specific activation in nitrogen metabolism, neural signalling, glucose metabolism, or energy metabolism between the two groups. The radar plot (Figure 3B) further indicated that glucose was the most contributing metabolite among nominally associated candidate metabolites, with a VIP value of 1.55 and log_2_fold-change of 0.76. This observation confirms the downregulation of glucose in the SC-LW group as shown in the heatmap. The correlation heatmap reveals descriptive modular co-variation patterns among these nominally associated candidate metabolites (Figure 3C). Note that these pairwise correlation patterns are derived from *n* = 12 samples and should be interpreted with caution. A cluster of metabolites centred on glucose, lactic acid, and the TCA cycle intermediate 2-ketoadipate exhibited continuous dark-red blocks, forming a highly positively correlated network. This suggests that energy-generating pathways, such as glycolysis and the tricarboxylic acid cycle, exhibit covariation across samples and may be associated with variation in overall metabolic phenotype. These correlative patterns cannot establish causal relationships. A distinct dark blue block was observed between L-glutamic acid and 4-aminobutyric acid, indicating a negative correlation between these excitatory and inhibitory neurotransmitter precursors. Such a correlation only reflects covariation and cannot directly confirm biological interaction.

#### 3.2.3. Kegg-Pathway Mapping Analysis of Nominally Associated Candidate Metabolites

KEGG pathway mapping was performed on nominally associated candidate metabolites identified from a skin metabolomic comparison between the two maternal families exhibiting LC-SW and SC-LW-related phenotypes. These nominally associated candidate metabolites were mapped to multiple functional KEGG pathways (Supplementary Table S2). The top five KEGG pathways ranked by the number of mapped candidate metabolites were: Metabolic pathways, Biosynthesis of amino acids, ABC transporters, Central carbon metabolism in cancer (KEGG term representing metabolic features of highly proliferative cells; no pathological cancer status is implied), and Aminoacyl-tRNA biosynthesis. It should be noted that these KEGG pathway identifiers are database-derived annotations. Mapping of candidate metabolites to these entries does not directly prove that the corresponding biological programmes are functionally activated within cashmere-goat skin follicle tissue.

#### 3.2.4. Database Derived Metabolite Reaction Pathway Association Network of Nominally Associated Candidate Metabolites

**Figure 4:**
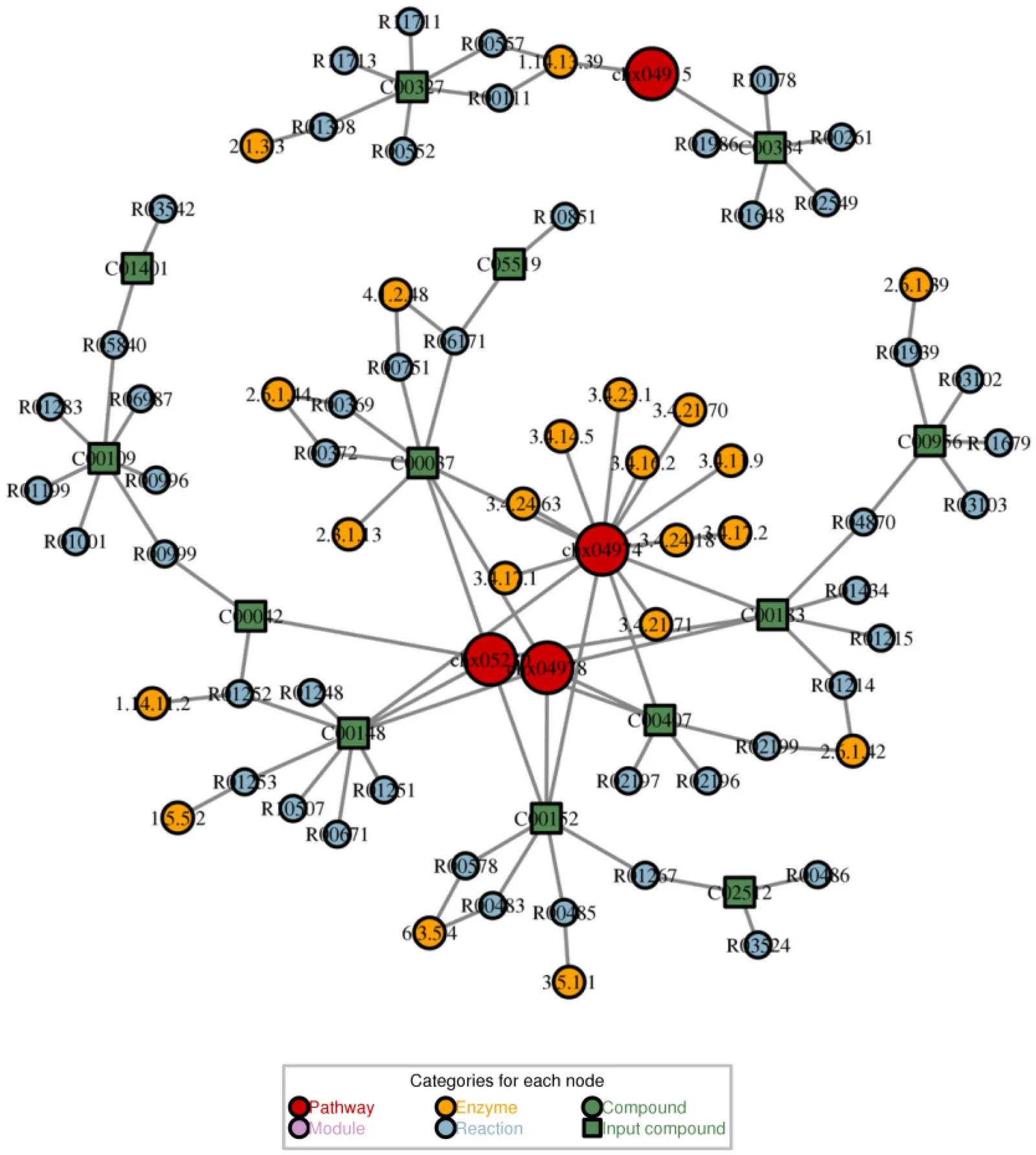
Database derived metabolite reaction pathway association network. This network is built based on public KEGG database annotations, without experimental measurement of enzymes or regulatory proteins. Red circles represent metabolic pathways; yellow circles represent enzyme entries retrieved from the KEGG database (these enzymes were not experimentally quantified in the present study); green circles represent background metabolites from database records; purple circles represent database-defined molecular modules; blue circles represent biochemical reactions extracted from KEGG; green squares correspond to nominally associated candidate metabolites identified in our comparison.

Nominally associated candidate metabolites including alanine, L-allothreonine, 2-ketobutyric acid, succinic acid, proline, glucose, alpha aminoadipic acid, valine, isoleucine, asparagine, and 3-cyanoalanine were connected to reaction and enzyme entries retrieved from the public KEGG database, including meprin B, pancreatic elastase II, glycolaldehyde + 2-oxobutanoate + alanine, O-succinyl-L-homoserine succinate-lyase, and L-2-aminohexanedioate: L-cysteine. These database-annotated connections are primarily associated with the pathways of Protein digestion and absorption, Mineral absorption, and Central carbon metabolism in cancer. The candidate metabolites L-citrulline and 4-aminobutanoate are associated with the database entry nitric-oxide synthase (NADPH), and this database link points toward the Estrogen signalling pathway (Figure 4). We emphasise that all enzymes shown in this network originate solely from KEGG database annotation and were not experimentally measured in the current work.

## 4. Discussion

It should be emphasised that the metabolic differences observed in this study derive from comparison between two specific maternal families under farm conditions. Owing to confounding between maternal family background and cashmere-related traits, we cannot definitively attribute all observed metabolic variation solely to differences in cashmere traits.

In this study, untargeted GC-TOF-MS metabolomics was used to capture widespread metabolite differences in skin tissue between the two goat groups. It should be noted that the current study focuses on untargeted skin metabolomics and does not include genomic marker-based population genetic analysis. Metabolomic profiles represent downstream intermediate molecular phenotypes shaped by genetic and physiological status. Our metabolomic findings provide a functional molecular reference resource for future genetic studies exploring the causal genetic variants underlying variation in cashmere traits in Liaoning cashmere goats.

Metabolomics, as an important branch of systems biology, reflects the global, dynamic changes in small-molecule metabolites within an organism under specific physiological conditions. It is particularly suitable for dissecting complex economic traits such as cashmere quality, which are regulated by multiple genes and pathways [11]. Compared with traditional phenotypic measurements or single-gene studies, metabolomics provides a functional readout closer to the phenotypic endpoint. In this study, multivariate PCA and OPLS-DA models, supported by model diagnostic indices and permutation test results, indicated suggestive metabolic separation between the two phenotypic groups. These multivariate modelling results are exploratory observations and do not constitute definitive statistical proof; independent validation in additional animal cohorts is needed to verify systematic metabolic differences further. This finding supports the close relationship between metabolic phenotype and genetic background and provides evidence for understanding differences in cashmere quality from a metabolic perspective [12].

Pearson correlation analysis revealed co-variation among the abundances of several key metabolites involved in glycolysis and the TCA cycle-related pathways. Such covariation patterns reflect metabolite interrelationships among sampled individuals but do not directly demonstrate pathway activation, altered metabolic flux, or causal effects. These metabolite patterns may reflect potential differences in energy-related metabolic profiles in skin follicle cells of LC-SW goats. Direct conclusions regarding cellular metabolic activity cannot be drawn solely from metabolite abundance data. Hair follicles, as highly active miniature organs, require large amounts of ATP and reducing equivalents for their cyclic growth, including the rapid synthesis phase of cashmere fibres [13]. Altered abundance of glucose and TCA cycle intermediates in LC-SW may provide a candidate correlative clue linking energy-related metabolism to longer cashmere fibre and higher cashmere yield. We cannot infer actual metabolic flux from untargeted metabolomics data alone. This finding is consistent with observations from studies on high-quality wool, where high-yield wool traits are associated with a higher flux of glucose metabolism in the skin tissue [14].

Among the nominally associated candidate metabolites, various amino acids and their related metabolites, including valine, isoleucine, proline, glycine, and asparagine, were significantly upregulated in the LC-SW group. KEGG pathway mapping analysis also showed pathways such as “Biosynthesis of amino acids” and “Aminoacyl-tRNA biosynthesis”. The main component of cashmere fibre is keratin, a type of hard protein rich in specific amino acids such as cysteine, proline, and glycine [15]. Keratinocytes in the skin follicle require a large and appropriately proportioned supply of amino acids to synthesise keratin. Our metabolite-level observations show a higher relative abundance of multiple amino acid-related compounds in LC-SW skin. These changes in abundance cannot directly prove enhanced biosynthesis or transport capacity at the functional level. In particular, proline and glycine are not only components of keratin but are also involved in maintaining the elasticity and structural stability of the fibre [16]. Therefore, the strengthening of the amino acid metabolic network is another important metabolic feature associated with improved cashmere quality traits.

Among the nominally associated candidate metabolites, lipid-related metabolites such as behenic acid, lignoceric acid, and arachidic acid, as well as Xylitol (a sugar alcohol metabolite involved in carbohydrate and polyol metabolism), were also altered. The upregulation of long-chain fatty acids, such as behenic acid, together with KEGG pathway mapping of the “Biosynthesis of unsaturated fatty acids” pathway, suggests that lipid metabolism may also play a role in regulating cashmere quality. Skin lipids are an important component of the hair follicle stem cell microenvironment and also contribute to the lipid layer on the fibre surface, thereby affecting its lustre, softness, and hydrophobicity [17]. In addition, certain fatty acids may act as signalling molecules to regulate inflammatory responses and hair-follicle cycling [18]. However, the specific mechanisms by which lipid metabolism contributes to cashmere quality formation still require further in-depth investigation using lipidomics approaches.

We observed differences in the relative abundances of glutamate and GABA between the two maternal families. These changes in abundance represent correlational variation in metabolite profiles. Further dedicated functional experiments would be required to explore their potential biological roles. GABA is the main inhibitory neurotransmitter in the central nervous system, while Glu is the main excitatory neurotransmitter. Both are also expressed in the skin and are involved in regulating hair follicle cycling, inflammation, and cell proliferation [19]. Multiple candidate metabolites were mapped to the “GABA ergic synapse” and “Neuroactive ligand receptor interaction” pathways in our KEGG mapping results, suggesting the potential importance of neurotransmitter-related metabolism in the local skin environment. The downregulation of these two metabolites in the SC-LW group correlates with its shorter cashmere phenotype, hinting at a possible link between neurotransmitter-related metabolism and cashmere growth duration; this hypothesis requires further functional validation. These findings provide some direction for future research.

Through KEGG pathway mapping and correlation network analysis, this study not only identified individual pathways but also preliminarily outlined a framework involving the coordinated action of multiple pathways. “Metabolic pathways”, the most broadly mapped category, serves as the central platform integrating energy, amino acid, and lipid metabolism. The ABC transporters pathway, to which multiple candidate metabolites were mapped, highlights the potential importance of transmembrane transport systems in efficiently moving nutrients (such as amino acids and sugars) from the blood into hair follicle cells [20,21]. Multiple nominally associated candidate metabolites were mapped to the “central carbon metabolism in cancer” pathway; this observation does not reflect pathological cancer conditions in goat skin. This is a standard KEGG database annotation describing metabolic reprogramming for fast-dividing cells. During the anagen phase, hair follicle cells undergo vigorous proliferation and macromolecule synthesis, exhibiting high glycolysis and glutamine-driven carbon metabolism that matches the metabolic signature defined within this KEGG pathway. Similar mapping of this pathway has also been reported in previous omics studies of skin and hair follicles in domestic animals [22]. These pathways do not operate in isolation but are interconnected through shared key metabolites such as succinic acid and alpha-ketoglutarate. The findings of this study are similar to those from studies on wool quality in sheep. For example, in Merino sheep, wool diameter has been associated with levels of certain amino acids and energy metabolites in the skin [23]. Compared with previous studies focusing on individual genes or hormones, the metabolic landscape provided by this study helps to connect fragmented knowledge. For instance, previous research has shown that the insulin-like growth factor (IGF) system affects cashmere growth in goats [24], and IGF signalling is closely related to cellular glucose and amino acid uptake and utilisation. The upregulation of glucose and amino acid metabolism observed in this study may be a downstream effect, thereby bridging the gap in understanding how genetic factors ultimately influence the phenotype through metabolic pathways.

Although this study has yielded some modest findings, several limitations remain, which also point to future research directions for the research group:

(1) All experimental animals were sourced from a single farm, and the phenotypic and metabolomic differences observed may not be generalised to all Liaoning cashmere goat populations. Expanded sampling across multiple farms and geographical regions is needed for external validation. Second, the non-inbred status of experimental animals was assessed by farm-maintained three-generation pedigree records rather than genome-wide SNP-derived relatedness or inbreeding coefficients. Future genomic analyses would help precisely evaluate kinship within and between families. Third, the sample size for skin metabolomic analysis was relatively small (*n* = 6 per family). Larger sample cohorts are required to confirm the identified metabolic signatures. Fourth, metabolomic analysis reveals correlative relationships; further functional experiments are necessary to clarify the causal effects of key nominally associated candidate metabolites on cashmere-related traits. Most importantly, phenotype and maternal family genetic background are confounded in our experimental design: the LC-SW-related phenotype is predominantly found in one maternal family, and the SC-LW-related phenotype in the other. Therefore, the observed metabolic divergence represents a comparison between these two particular farm-kept maternal families, rather than universal mechanistic evidence broadly applicable to all LC-SW and SC-LW Liaoning cashmere goat subtypes. Independent animal materials from additional unrelated families are required in future work to disentangle family background effects from cashmere phenotype-driven metabolic variation.

(2) Our metabolomic analysis is based on group-level comparison of two maternal families. level comparison of two maternal families. Owing to the small metabolomic sample size (*n* = 12 total), we could not reliably evaluate associations between metabolite abundance and individual-level continuous cashmere quality traits. Direct metabolite- trait correlation requires larger sample cohorts in future investigations.

(3) Metabolomic analysis primarily reveals correlations. Whether the identified nominally associated candidate metabolites are causes, consequences, or merely correlates of cashmere quality differences remains to be functionally validated. For example, hair follicle cells could be cultured in vitro, and key metabolites such as GABA, glutamate, or specific amino acids could be added or inhibited to observe their effects on cell proliferation, differentiation, and keratin gene expression.

(4) Metabolism is the endpoint of gene expression. Metabolomic data alone are insufficient to trace the upstream regulatory mechanisms. Future studies should integrate transcriptomic, proteomic, and even epigenomic data with phenotypes to identify key regulatory genes (such as those encoding metabolic enzymes or transporters).

(5) Pairwise metabolite correlation analysis was performed on only 12 metabolomic samples; correlation coefficients are prone to sampling fluctuation, and these co-variation patterns are exploratory and require validation in larger cohorts.

## 5. Conclusions

Our study demonstrates distinct metabolic profiles associated with cashmere-related phenotypic differences between the two groups. We identified correlative changes in skin amino-acid, carbohydrate and polyol metabolites in two Liaoning cashmere-goat maternal families, offering exploratory molecular clues for cashmere-quality research. Because phenotypic variation is confounded with maternal family genetic background and the metabolomic sample size is limited, these associations cannot be generalized to all LC-SW and SC-LW subtypes, and causal links cannot be concluded from the present data. Larger independent cohorts and functional experiments are needed to validate candidate metabolites and clarify their biological roles in cashmere trait formation.

## Figures and Tables

**Table 1:** Comparison of Phenotypic Traits of Cashmere Fibers.

Maternal Family	Body Weight (kg)	Cashmere Yield (g)	Fiber Length (cm)	Fiber Diameter (μm)
LC-SW	31.45 ± 2.04 ^a^	670.20 ± 98.17 ^a^	6.72 ± 1.03 ^a^	15.67 ± 1.21 ^a^
SC-LW	28.04 ± 1.98 ^b^	523.70 ± 89.24 ^b^	5.48 ± 1.11 ^b^	15.24 ± 0.98 ^a^
*p*-values	0.018	0.026	0.031	0.427

LC-SW: Long-cashmere-short-wool Type. SC-LW: Short-cashmere-long-wool Type. Note 1: Comparison of cashmere-quality-related phenotypes between two maternal families. Phenotypic measurements were performed on 120 goats (LC-SW: *n* = 60; SC-LW: *n* = 60). An independent-samples *t*-test was used to compare groups. Data are presented as mean ± standard deviation. A subset of 6 animals from each family (total *n* = 12) was randomly selected from the 120 phenotyped individuals for untargeted skin metabolomics analysis. Note 2: Columns with different lowercase letters indicate significant differences (*p* < 0.05), while those with the same letter indicate no significant differences (*p* > 0.05). *p*-values shown in the last column are derived from Student’s *t*-test comparing LC-SW versus SC-LW groups. Phenotypic data in this table were obtained from goats sampled at the Wafangdian Animal Husbandry Extension Centre. Further sampling of additional goat herds from different farms is required to verify the universality of these phenotypic differences.

## Data Availability

The data reported in this paper have been deposited in the Figshare (http://doi.org/10.6084/m9.figshare.26886394).

## Supplementary Materials

The following supporting information can be downloaded at: https://www.mdpi.com/article/10.3390/genes17101298/s1; Table S1: Nominally associated candidate metabolites; Table S2: KEGG-pathway mapping of candidate differential metabolites.
