# Supplementary material for: Metabolomic Profiling Screening for Molecular Level Associations Related to Cashmere Quality Related Phenotypic Types in Liaoning Cashmere Goats
Source: Genes (Basel). 2026 Oct 18;17(10):1298. doi: 10.3390/genes17101298 (PMC13704026; doi:10.3390/genes17101298)
Supplement: Supplementary file 1 [file genes-17-01298-s001.zip › Supplementary Table S1.pdf]

Table S1. Nominally associated candidate metabolites.

NAME	MSI	FOLD CHANGE	P-VALUE	Q-VALUE	VIP
glucose 2	2	0.803689372	0.019039487	0.090495629	1.552086809
proline	2	1.338055714	0.023199851	0.098223383	1.456498667
valine	2	1.560285445	0.000229593	0.006652583	2.040557821
glycine 2	2	1.225101783	0.005366423	0.043906856	1.681742462
succinic acid	2	1.694645696	0.000955751	0.013446723	1.91779821
alanine 1	2	1.217008342	0.020735481	0.093863852	1.500960533
Isoleucine	2	1.584365013	0.000361441	0.007955487	2.026589234
oxoproline	2	1.150210153	0.029683735	0.110589855	1.41549286
benzoic acid	2	1.334102108	0.001315365	0.01631916	1.913091215
L-Allothreonine 1	2	1.273836805	0.001301433	0.016216105	1.854093966
lactic acid	2	1.637404125	5.02792E-06	0.001434721	2.138685296
Ethanolamine	2	1.252817029	0.04208783	0.130819075	1.34702919
serine 1	2	1.354141691	0.00020299	0.006282899	1.991633952
mannose 2	2	0.835386945	0.012852332	0.074715457	1.605801211
cellobiose 2	2	1.501268072	0.033830029	0.11716734	1.441669377
unknown	2	0.713684628	0.000109098	0.004447329	2.039229663
phenylalanine 1	2	1.628654801	0.016082942	0.083741749	1.563329041
Lignoceric acid	2	1.269090828	0.041217589	0.129453888	1.368872664
unknown	2	0.734979868	0.031082461	0.112917267	1.427604368
3-Cyanoalanine	2	1.733848494	0.000421845	0.008363929	1.911239807
4-aminobutyric acid 1	2	0.694296327	0.001033887	0.014048604	1.978717617
Arachidic acid	2	1.169909703	0.020115916	0.092671249	1.490790505
Behenic acid	2	1.521796014	0.000469951	0.008635493	1.955937638
glycolic acid	2	1.177699081	0.00306421	0.031086616	1.747318125
xylitol	2	1.364283185	0.027778753	0.107221969	1.511480214
alpha-Aminoadipic acid	2	1.964654395	0.042266761	0.131096278	1.457573201
asparagine 4	2	2.197171569	0.00445568	0.039477624	1.680620682
malonic acid 1	2	1.106042562	0.023495318	0.098708483	1.490212548
L-glutamic acid	2	0.676528359	0.000615304	0.010236088	1.957843609
unknown	2	1.276809081	0.0123444	0.073101435	1.821370548
2-ketoadipate 2	2	4271.651403	0.032497015	0.115156022	1.600026104
unknown	2	1.150951624	0.000489877	0.00873667	1.968165915
citrulline 2	2	1.884039215	0.014744091	0.080239875	1.2958866
unknown	2	0.238473671	0.040121529	0.127693278	1.494318079
unknown	2	1.211671809	0.011926349	0.071727137	1.559884581
unknown	2	3.766246599	0.013176906	0.075716236	1.636346757
D-erythronolactone 2	2	0.907730429	0.023259508	0.098321932	1.53832381
Dihydroxyacetone	2	3.455287926	0.00039053	0.008162847	2.200210979
D-galacturonic acid 2	2	0.811551607	0.023285904	0.09836544	1.481062598
N-Ethylglycine 1	2	1.137109094	0.004026029	0.03712277	1.759959003
prostaglandin A2 1	2	8.714757038	8.11746E-05	0.003860538	1.673408299
unknown	2	1.323167969	0.020471201	0.093360249	1.491526338
hydroxylamine	2	1.209363288	0.019026196	0.090467903	1.688425137
unknown	2	1.18393947	0.000428282	0.008402727	1.948108405
unknown	2	2.803229687	0.006061876	0.047247278	1.444074094
unknown	2	1.941352718	5.85515E-05	0.003635743	2.218294164
Androstanediol	2	7.869824651	0.004906822	0.041759844	1.639057261
2-ketobutyric acid 2	2	1.133260188	0.002840667	0.029506253	1.794936804

Methyl Palmitoleate	2	0.868859226	0.011667694	0.070855162	1.676539026
unknown	2	1.71751122	3.51291E-05	0.00331702	2.08044721
Aminooxyacetic acid	2	1.13967827	0.047019519	0.139761052	1.351673179
5-hydroxytryptophan	2	10.06448976	0.007718901	0.055064879	1.552990954
unknown	2	1.357110998	0.000403828	0.008250839	1.981368474
unknown	2	1.196188097	0.010084545	0.065124362	1.553554504
Analyte 10	2	0.597390623	0.006219714	0.047967593	1.730314961
Analyte 11	2	1.456179464	4.82668E-05	0.003527425	2.092638069
Analyte 18	2	1.225816362	0.023539779	0.098780833	1.638085274
Analyte 20	2	1.163134167	0.010465295	0.066568008	1.675637694
Analyte 25	2	1.216830009	0.036016078	0.120772349	1.425246144
Analyte 29	2	0.236499444	0.03568904	0.120229088	1.494859933
Analyte 30	2	1.133588707	0.028848735	0.109142914	1.496202916
Analyte 31	2	3.149186088	0.01800706	0.088275148	1.614844059
Analyte 32	2	1.127721081	0.01702513	0.086029946	1.580713914
Analyte 37	2	1.142536571	0.044749856	0.135616011	1.380135691
Analyte 58	2	1.113703221	0.040059063	0.127591522	1.400819636
Analyte 70	2	1.133549658	0.015934549	0.08336861	1.560696072
Analyte 99	2	1.108671651	0.034266165	0.117805789	1.438476889
Analyte 103	2	1.101386215	0.032772439	0.11557919	1.475567806
Analyte 116	2	1.107376991	0.028466844	0.108466113	1.452069735
Analyte 143	2	1.148145194	0.046022896	0.137960687	1.281483163
Analyte 151	2	28.3985048	0.017954327	0.088157984	1.708154894
Analyte 205	2	2.710912038	6.45745E-05	0.003685279	2.217990144
Analyte 248	2	1.182419848	0.007178944	0.052634744	1.712727276
Analyte 287	2	1.238501366	0.003790974	0.035751492	1.761218318
Analyte 303	2	1.21070135	0.018042276	0.088353181	1.491865209
Analyte 334	2	3.076572664	0.020274525	0.092980558	1.647838477
Analyte 349	2	1.364058395	0.004543266	0.039935246	1.619929404
Analyte 390	2	351.7413919	0.036771051	0.122007579	1.677581976
Analyte 407	2	1.314061529	0.02347944	0.098682604	1.606999252
Analyte 437	2	0.154573	0.007675359	0.054873792	1.533635058
Analyte 549	2	1.184827136	0.030601949	0.112130901	1.441142459
Analyte 561	2	0.372321673	0.012952235	0.075025981	1.370535228
Analyte 571	2	155.9337452	0.026027282	0.103903971	1.595151949
Analyte 577	2	0.001929599	0.00540858	0.044095516	1.963618537
Analyte 595	2	0.435251001	0.033508384	0.116690401	1.327693264
Analyte 619	2	0.864245182	0.029626147	0.110491481	1.473796616
Analyte 638	2	1027.967525	0.000874664	0.012775326	2.297809395
Analyte 663	2	0.560489978	0.005291908	0.043570102	1.735261804
Analyte 690	2	0.784220718	0.002730002	0.028695657	1.779462346
