# Supplementary material for: Metabolomic Profiling Screening for Molecular Level Associations Related to Cashmere Quality Related Phenotypic Types in Liaoning Cashmere Goats
Source: Genes (Basel). 2026 Oct 18;17(10):1298. doi: 10.3390/genes17101298 (PMC13704026; doi:10.3390/genes17101298)
Supplement: Supplementary file 1 [file genes-17-01298-s001.zip › Supplementary Table S2.pdf]

Table S2. KEGG-pathway mapping of candidate differential metabolites.

Description	compounds (dem)	Compounds (dem) Metabolites	Expression mode
Metabolic pathways	23	proline	up
		valine	up
		glycine 2	up
		succinic acid	up
		alanine 1	up
		isoleucine	up
		oxoproline	up
		benzoic acid	up
		L-Allothreonine 1	up
		Ethanolamine	up
		3-Cyanoalanine	up
		4-aminobutyric acid 1	down
		glycolic acid	up
		xylitol	up
		alpha-Aminoadipic acid	up
		asparagine 4	up
		L-glutamic acid	down
		citrulline 2	up
		Dihydroxyacetone	up
		D-galacturonic acid 2	down
		prostaglandin A2 1	up
		hydroxylamine	up
		2-ketobutyric acid 2	up
Biosynthesis of amino acids	8	proline	up
		valine	up
		glycine 2	up
		isoleucinee	up
		alpha-Aminoadipic acid	up
		asparagine 4	up
		citrulline 2	up
		2-ketobutyric acid 2	up
ABC transporters	6	proline	up
		valine	up
		glycine 2	up
		isoleucine	up
		xylitol	up
		D-galacturonic acid 2	down
Central carbon metabolism in cancer	6	proline	up
		valine	up
		glycine 2	up
		succinic acid	up
		isoleucine	up
		asparagine 4	up
Aminoacyl-tRNA biosynthesis	5	proline	up
		valine	up
		glycine 2	up
		isoleucine	up
		asparagine 4	up
Protein digestion and absorption	5	proline	up
		valine	up
		glycine 2	up
		Isoleucine	up
		asparagine 4	up
Mineral absorption	5	proline	up
		valine	up
		glycine 2	up
		isoleucine	up
		asparagine 4	up
Carbon metabolism	4	glycine 2	up
		succinic acid	up
		glycolic acid	up
		Dihydroxyacetone	up
2-Oxocarboxylic acid metabolism	4	valine	up

Description	compounds (dem)	Compounds (dem)	
		Metabolites	Expression mode
		isoleucine	up
		alpha-Aminoadipic acid	up
		2-ketobutyric acid 2	up
		succinic acid	up
Alanine, aspartate and glutamate metabolism	3	4-aminobutyric acid 1	down
		asparagine 4	up
		glycine 2	up
Glycine, serine and threonine metabolism	3	L-Allothreonine c005519	up
		2-ketobutyric acid 2	up
		valine	up
Valine, leucine and isoleucine biosynthesis	3	Isoleucine	up
		2-ketobutyric acid 2	up
		glycine 2	up
Lysine degradation	3	succinic acid	up
		alpha-Aminoadipic acid	up
		proline	up
Arginine and proline metabolism	3	oxoproline	up
		4-aminobutyric acid 1	down
		glycine 2	up
Glyoxylate and dicarboxylate metabolism	3	succinic acid	up
		glycolic acid	up
		Lignoceric acid	up
Biosynthesis of unsaturated fatty acids	3	Arachidic acid	up
		Behenic acid	up
Pentose and glucuronate interconversions	2	xylitol	up
		D-galacturonic acid 2	down
Valine, leucine and isoleucine degradation	2	valine	up
		isoleucine	up
Phenylalanine metabolism	2	succinic acid	up
		benzoic acid	up
Propanoate metabolism	2	succinic acid	up
		2-ketobutyric acid 2	up
Butanoate metabolism	2	succinic acid	up
		4-aminobutyric acid 1	down
Nicotinate and nicotinamide metabolism	2	succinic acid	up
		4-aminobutyric acid 1	down
cAMP signaling pathway	2	succinic acid	up
		4-aminobutyric acid 1	down
Neuroactive ligand-receptor interaction	2	glycine 2	up
		4-aminobutyric acid 1	down
Synaptic vesicle cycle	2	glycine 2	up
		4-aminobutyric acid 1	down
Retrograde endocannabinoid signaling	2	Ethanolamine	up
		4-aminobutyric acid 1	down
GABAergic synapse	2	succinic acid	up
		4-aminobutyric acid 1	down

Note: (1)nominally associated candidate metabolites were screened by comparing skin metabolome of LC-SW versus SC-LW cashmere goats. “Up” represents metabolites significantly higher in LC-SW group; “Down” represents metabolites significantly higher in SC-LW group.

(2)Table shows the count of candidate differential metabolites mapped to each KEGG pathway entry, No statistical enrichment testing was performed.

(3)KEGG pathway names are database annotations only; they do not directly prove that the corresponding physiological programmes are active in skin-follicle tissue.
